# Atacicept in combination with MMF and corticosteroids in lupus nephritis: results of a prematurely terminated trial

**DOI:** 10.1186/ar3738

**Published:** 2012-02-07

**Authors:** Ellen M Ginzler, Stephen Wax, Anand Rajeswaran, Samuel Copt, Jan Hillson, Eleanor Ramos, Nora G Singer

**Affiliations:** 1 SUNY Downstate Medical Center, 450 Clarkson Avenue, Box 42, Brooklyn, NY 11203, USA; 2 EMD Serono Inc., 1 Technology Place, Rockland, MA 02370, USA; 3 Merck Serono S.A., Chemin des Mines 9, 1202 Geneva, Switzerland; 4 ZymoGenetics Inc., 1201 Eastlake Avenue, East Seattle, Washington 98102-3702, USA; 5 Case Western Reserve University, 2040 Adelbert Rd, Cleveland, OH 44106-7060, USA

**Keywords:** atacicept, clinical trial, immunoglobulin, lupus nephritis, mycophenolate mofetil, nephrotic-range proteinuria

## Abstract

**Introduction:**

Atacicept is a soluble, fully human, recombinant fusion protein that inhibits B cell-stimulating factors APRIL (a proliferation-inducing ligand) and BLyS (B-lymphocyte stimulator). The APRIL- LN study aimed to evaluate the efficacy and safety of atacicept in patients with active lupus nephritis (LN), receiving newly initiated corticosteroids (CS) and mycophenolate mofetil (MMF).

**Methods:**

This was a randomized, double-blind, placebo-controlled Phase II/III, 52-week study. At screening (Day -14), patients initiated high-dose CS (the lesser of 0.8 mg/kg/day or 60 mg/day prednisone) and MMF (1 g daily, increased by 1 g/day each week to 3 g daily). From Day 1, atacicept (150 mg, subcutaneously, twice weekly for 4 weeks, then weekly) was initiated with MMF along with a tapered dose of CS.

**Results:**

The trial was terminated after the enrollment of six patients, due to an unexpected decline in serum immunoglobulin G (IgG) and the occurrence of serious infections. Efficacy was thus not evaluated. By Day 1, serum IgG levels had declined substantially in patients then randomized to atacicept (*n *= 4) compared with placebo (*n *= 2). Patients receiving atacicept also had more severe proteinuria on Day -14 than those on placebo. Lymphocyte counts were low at screening in all patients. IgG decline continued following initiation (Day 1) of atacicept. Three atacicept-treated patients developed serum IgG below the protocol-defined discontinuation threshold of 3 g/l, two of whom developed serious pneumonia.

**Conclusions:**

Future studies are needed to characterize the safety, efficacy, and pharmacodynamic response of atacicept in LN patients.

**Trial Registration:**

ClinicalTrials.gov: NCT00573157

## Introduction

Current treatments for lupus nephritis (LN), a severe complication of systemic lupus erythematosus (SLE), may be associated with significant toxicity (for example, infertility, infection, malignancy), and complete remission rates remain low [1]. The pathogenesis of LN is complex and involves immune-mediated mechanisms, including autoantibody production and immune complex deposition in the kidney [2]. Atacicept is a soluble, fully human, recombinant fusion protein that inhibits the B cell-stimulating factors APRIL (a proliferation-inducing ligand) and BLyS (B-lymphocyte stimulator). APRIL and BLyS levels are altered in some autoimmune diseases associated with increased autoantibody production [3]. A Phase II/III randomized, double-blind, placebo-controlled clinical trial was initiated to evaluate the efficacy and safety of atacicept in patients with active LN who were receiving newly initiated immunosuppressive therapy with corticosteroids (CS) and mycophenolate mofetil (MMF). This trial was terminated after only six patients had been enrolled, as a result of unexpected falls in serum immunoglobulin G (IgG) and the occurrence of serious infections. No efficacy evaluation took place.

## Materials and methods

### Study design

On Study Day -14, patients commenced MMF (500 mg, twice daily, orally) and prednisone/prednisone equivalent (the lesser of 0.8 mg/kg/day or 60 mg/day, orally). MMF dose was increased to 1,000 mg twice daily at Day -7, thereafter up to a maximum of 1.5 g twice daily by Day 1. Day -14 to Day 1 was defined as the screening period. Patients fulfilling all entry criteria were randomized (1:1) on Day 1 to placebo or atacicept, 150 mg subcutaneously, twice weekly for 4 weeks, then 150 mg weekly for a planned 48 weeks. The CS dose was to be tapered starting at Week 5 by 5 mg/day/week, to 10 mg/day by Week 12. Assessments were scheduled post Day 1 at Weeks 2 and 4, then every 4 weeks to Week 52. Following the discontinuation of the study drug (completion of 52 weeks of treatment or early termination), follow-up visits were scheduled 4, 12 and 24 weeks after the last dose.

### Patients

Key inclusion criteria were: SLE diagnosis satisfying ≥ 4 of the 11 American College of Rheumatology criteria [4]; positive antinuclear antibody test (Hep-2 antinuclear antibody ≥ 1:80) and/or antibodies against double-stranded DNA ([anti-dsDNA] ≥ 30 IU/ml); renal biopsy within the 12 months preceding study entry, with histological findings consistent with active LN (International Society of Nephrology/Renal Pathology Society class III or IV LN); active LN, defined by proteinuria (urine protein:creatinine ratio [UPr:Cr] > 1.0 mg/mg) and hematuria (> 10 red blood cells [RBCs]/high-powered field [hpf] with or without RBC casts): all other causes of hematuria of non-glomerular origin were excluded. Key exclusion criteria were: renal disease unrelated to SLE; calculated glomerular filtration rate ([GFR] based on the Modification of Diet in Renal Disease equation) ≤ 30 ml/min/1.73 m^2 ^at screening; comorbidities requiring CS; progressive disease (25% decrease in GFR or doubling of UPr:Cr between screening and randomization). The Institutional Review Board or Ethics Committee at each center approved the study; written informed consent was obtained from each patient or their legal representative, according to the Declaration of Helsinki.

### Criteria for discontinuation

Key criteria for discontinuation were: 25% decrease in calculated GFR or doubling of UPr:Cr compared with baseline; IgG level < 3 g/L; minimal or no improvement in renal parameters at Week 24.

## Results

### Patient disposition and safety results

At the time of termination, six patients from four centers in the US had been randomized: two to receive placebo and four to receive atacicept (Table 1). Four patients were women and two men; the age range was 18 to 54 years.

**Table 1:** Demographic characteristics, disease history, duration on study and reasons for discontinuation

Patient #	Race	**Disease history**^a^			Prior therapy		Study drug treatment duration (Days)	Reason for discontinuation
				
		SLE (Months)	LN (Months)	Prior renal flare	MMF	CTX		
2	African American	38	< 1	-	-	-	148	Leukocytoclastic vasculitis
8 3	Caucasian	46	38	+	+	-	85	Study termination
	African American	< 1	< 1	-	-	-	29	*H. influenzae *pneumonia, IgG < 3 g/l
5	African American	3	< 1	-	-	-	230	Study termination
13	African American	52	52	+	+	+	31	IgG < 3 g/l^b^
14	Asian	32	< 1	+	+	+	18	IgG < 3 g/l

Patients' mean (SD) height and weight were 169.3 (9.7) cm and 83.3 (20.8) kg, respectively. Patients #2 and #8 randomized to placebo; Patients #3, #5, #13 and#14 randomized to atacicept 150 mg subcutaneously twice weekly (4 weeks), then 150 mg weekly (48 weeks). ^a^Disease history: time between SLE/LN diagnosis and consent. All patients had Class IV renal disease. ^b^Patient #13 diagnosed with severe *Legionella *pneumonia on Day 34, 1 day after study discontinuation. CTX, cyclophosphamide; IgG, immunoglobulin G; LN, lupus nephritis; MMF, mycophenolate mofetil; SLE, systemic lupus erythematosus.

The courses of the four atacicept-treated patients are described here. Three patients developed serum IgG levels below the protocol-defined discontinuation criterion. Patient #3 had an IgG level of 2.9 g/l on Day 29 and developed *Haemophilus influenzae *pneumonia complicated by empyema, septicemia and pneumothorax; treatment was discontinued on Day 30. Patient #13 had an IgG level of 2.5 g/l on Day 30; treatment was discontinued on Day 33 and the patient was diagnosed with *Legionella pneumophila *pneumonia on Day 34. Patient #14 had an IgG of 2.5 g/l and was discontinued on Day 18. At Week 12 of the safety follow-up period, this patient developed *Bacillus *bacteremia. Patient #5 had atacicept administered until Day 230, when the study was discontinued. Her IgG level decreased from a baseline of 20.1 to 4.7 g/l at Week 12, and then gradually increased to 6.9 g/l at approximately Week 34 (when atacicept was terminated along with the study).

In both placebo-treated patients, IgG levels fell between Days 1 and 15, declining to nadirs at Weeks 4 and 8 above the lower limit of normal [LLN] (11.9 g/l in Patient #2; 10.2 g/l in Patient #8), before gradually increasing. Patient #2 discontinued study participation due to leukocytoclastic vasculitis, 148 days post randomization. Patient #8 continued until the time of study termination, 85 days post randomization.

The study was terminated early as a result of the above safety events and, thus, none of the patients reached full treatment to Week 52. Enrollment ceased and study drug administration stopped in all patients. All patients completed a 24-week post-treatment follow-up period. Table 1 provides demographic and disease history characteristics, study treatment duration, and reasons for discontinuation.

### Laboratory measurements

**Figure 1:**
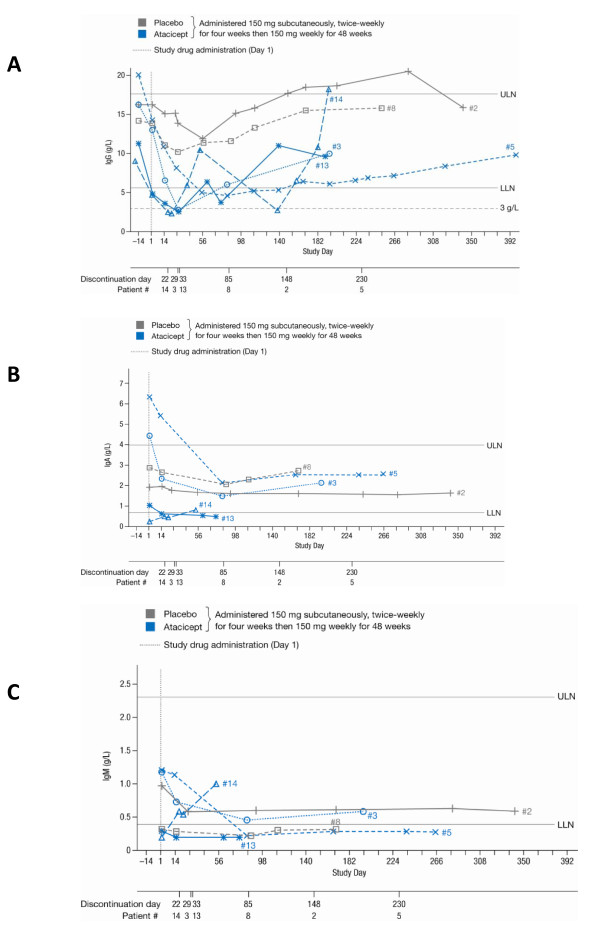
**Immunoglobulin (Ig) levels in all six patients, across the study period**. **A**) IgG, reference range (RR) 5.65 to 17.65 g/l, < 3 g/l represented study discontinuation threshold; **B**) IgA, RR 0.7 to 4 g/l; **C**) IgM, RR 0.4 to 2.3 g/l. IgA and IgM were not measured during the screening period (Days-14 to 1). Discontinuation days were not necessarily scheduled follow-up days. LLN, lower limit of normal; ULN, upper limit of normal.

Immunoglobulin levels across the study period are shown in Figure 1. Serum IgG levels were normal or elevated in all patients at screening. Following the initiation of MMF and CS, a marked reduction in IgG (up to 57%) was observed by Day 1 (Table 2; Figure 1A). The decreases were largest in those patients who were subsequently randomized to atacicept. Serum IgG levels continued to decline between Days 1 and 14, by up to 47% versus Day 1 and 72% versus Day -14 (Table 2). Neither IgA nor IgM levels showed a consistent decrease among enrolled patients (Table 2; Figures 1B, C).

**Table 2:** Renal parameters, immunoglobulin (Ig)A, IgG and IgM levels, and IgG course, Days-14 to 14

Patient #	Screening day (Day -14)*			Day 1						Day 14					
	
	GFR	UPr:Cr	IgG	GFR	UPr:Cr	IgA	IgM	IgG	IgG change from Day -14	GFR	UPr:Cr	IgA	IgM	IgG	IgG change from Day 1
	
	(ml/min/1.73 m^2^)	(mg/mg)	(g/l)	(ml/min/1.73 m^2^)	(mg/mg)	(g/l)	(g/l)	(g/l)		(ml/min/1.73 m^2^)	(mg/mg)	(g/l)	(g/l)	(g/l)	(%)
2	73.5^L^	1.6	16.3	68.4^L^	0.2	1.9	1.0	16.3	**0**	45.3^L1, a^	0.1^a^	2.0^a^	0.8^a^	15.1^a^	**-7.4**
8	78.7	2.3	14.2	76.3	N/A	2.9	0.3^L^	13.9	**-2.1**	88.8^a^	0.8^a^	2.7^a^	0.3^L, a^	11.1^a^	**-20.1**
3	73.4^L^	8.2	16.3	81.6	5.6	*4.5*	1.2	13.0	**-20.3**	72.2^L^	8.1^a^	2.3^a^	0.7^a^	6.5^a^	**-49.7**
5	74.5^L^	3.0	*20.1*	89.0	1.0	*6.3*	1.2	14.3	**-28.9**	69.7^L, b^	7.1	*5.4*^b^	1.1^b^	10.9^b^	**-23.8**
13	33.6^L2^	4.6	11.3	30.9^L2^	12.4	1.1	0.3^L^	4.9^L^	**-56.9**	34.1^L2, a^	8.4^a^	0.6^L, a^	< 0.2^L^	3.6^L, a^	**-25.5**
14	26.9^c,^†^, L2^	12.0	9.1	24.4^L2^	18.0	< 0.3^L^	< 0.2^L^	4.8^L^	**-47.1**	38.8^L2, d^	9.0^d^	0.5^L, d^	0.6^d^	2.5^L, d^	**-47.4**

Patients #2 and #8 randomized to placebo; Patients #3, #5, #13 and #14 randomized to atacicept 150 mg subcutaneously twice weekly (4 weeks), then 150 mg weekly (48 weeks). *IgA and IgM levels not recorded at screening; GFR, glomerular filtration rate; Ig, immunoglobulin; UPr:Cr, urine protein:creatinine ratio.

Values > ULN indicated in italics; ^L^Values < LLN with (Grade 1 to 4), where applicable; Ig reference ranges: IgA, 0.7 to 4.0 g/l; IgM, 0.4 to 2.3 g/l; IgG, 5.7 to 17.6 g/l. ^a^Measured Day 15; ^b^Measured Day 13; ^c^Measured Day -18; ^d^Measured Day 18. †GFR > 30 ml/min/1.73 m^2 ^on repeat testing.

At screening, the patients subsequently randomized to atacicept presented with more severe proteinuria (UPr:Cr ≥ 3.0 versus ≤ 2.3 mg/mg) and a lower mean GFR (52.1 versus 76.1 ml/min/1.73 m^2^) compared with those who were randomized to placebo on Day 1. Baseline GFR was particularly low in Patients #13 and #14, and remained so on Days 1 and 14. Whereas UPr:Cr had fallen below 1.0 mg/mg in Patient #2 on Days 1 and 14, and in Patient #8 on Day 14 (Day 1 reading unavailable), it remained high on Days 1 and 14 in patients randomized to atacicept (range 5.6 to 18.0 mg/mg, with the exception of a value of 1.0 mg/mg in Patient #5 on Day 1). At the end of the study period, both placebo-treated and two atacicept-treated patients (Patients #5 and #13) had UPr:Cr ratios that were lower than baseline values. In a third atacicept-treated patient (Patient #3), the ratio was approximately the same as at baseline, and in Patient #14 it was higher than at baseline.

Patients entered the study with wide variations in counts of white blood cells (WBCs), absolute neutrophils, and absolute lymphocytes (Table 3). During the study, WBCs fluctuated in all patients but were within the normal range by the 24-week follow-up visit in all except Patient #5. Neutrophilia was common at screening and Day 1. Lymphocyte concentrations at screening were below or at the lower end of the reference range (0.9 to 4.3 × 10^9^/l) in all patients (Table 3). In Patients #3 and #13, lymphocyte counts declined from a baseline (Day -14) level within the reference range (0.9 and 1.5 × 10^9^/l, respectively) to Grade 2 lymphopenia by Day 1 (0.7 × 10^9^/l in both). After addition of atacicept, lymphocytes continued to decline in both patients, with Grade 3 lymphopenia developing in Patient #3 on Day 29 (0.5 × 10^9^/l), coincident with *H. influenzae *pneumonia, and in Patient #13 on Days 15 and 30, the latter (0.5 × 10^9^/l) coincident with development of *L. pneumophila *pneumonia. CD4^+ ^counts (not measured at screening) were also low on Day 1, in all patients measured.

**Table 3:** Hematologic and serologic parameters Day -14 to end of treatment (EOT)

Patient #	White blood cells **(**× 10^9^/l**)** RR 3.9-10.7				Absolute lymphocytes **(**× 10^9^/l**)** RR 0.9-4.3				Absolute neutrophils **(**× 10^9^/l**)** RR 2.0-7.2			
	
	Day -14	Day 1	Day 14 (± 1)	EOT	Day -14	Day 1	Day 14 (± 1)	EOT	Day -14	Day 1	Day 14 (± 1)	EOT
2	*11.2*	6.0	8.3	3.8	1.7	1.6	1.7	2.0	*8.9*	4.1	6.4	1.6^L1^

8	6.3	*15.1*	*12.4*	8.1	0.8^L2^	0.9	1.3	1.0	5.0	*13.7*	*10.6*	7.0

3	5.7	*11.1*	8.1	4.7	0.9	0.7^L2^	0.5^L2^	0.2^L3^	4.4	*10.1*	*7.4*	4.3

5	2.8^L2^	*15.6*	N/A	N/A	1.0	3.4	N/A	N/A	1.6^L1^	*10.6*	N/A	N/A

13	*14.7*	5.6	8.5	7.6	1.5	0.7^L2^	0.5^L3^	0.7^L2^	*13.1*	4.7	*7.8*	6.5

14	*15.5*^a^	*15.2*	8.3^b^	7.1	0.9^a^	1.8	0.8^L1, b^	0.8^L1^	*13.7*^a^	*12.9*	7.0^b^	5.7

	**CD4^+ ^(cells/μl) [%]** **RR 404-1612 [33-58] **				**Complement C3 (g/l)** **RR 0.9-1.8**				**Anti-dsDNA (IU/ml)** **< 30.0, negative; 30-75, borderline; > 75, positive**			
	
**Patient #**	**Day 1**	**Day 28 (± 2)**	**EOT**		**Day 1**	**Day 14 (± 1)**	**EOT**		**Day** **-14**	**Day 1**	**Day 14 (± 1)**	**EOT**

2	489 [25.8^L^]	*1731 *[34.1]	980 [44.6]		0.686^L^	0.904	0.721^L^		*917.2*	*375.7*	*168.1*	*463.9*

8	277^L2 ^[23.5^L^]^c^	273^L2 ^[29.7^L^]	195^L3 ^[24.0^L^]		0.915	1.150	1.070		*325.5*	*306.1*	*97.6*	20.1

3	N/A	56^L3 ^[13.7^L^]	45^L4 ^[21.4^L^]		0.533^L^	0.800^L^	0.906		*155.3*	*77.6*	37.5	< 12.3

5	529 [22.1^L^]	809 [30.1^L^]	603 [46.8]		1.030	1.630	1.700		56.7	43.0	34.3	12.6

13	240^L2 ^[38.7]	95^L3 ^[23.8^L^]	163^L3 ^[19.3^L^]		0.462^L^	0.717	0.909		*87.5*	26.0	13.0	12.7

14	135^L3 ^[19.1^L^]	N/A	158^L3 ^[19.9^L^]		0.434^L^	0.541^b^	0.609^L^		*349.6*	*88.1*	28.8^b^	20.1

Patients #2 and #8 randomized to placebo; Patients #3, #5, #13 and #14 randomized to atacicept 150 mg subcutaneously twice weekly (4 weeks), then 150 mg weekly (48 weeks). N/A, not available; RR, reference range. Values > ULN indicated in italics; ^L^Values < LLN, with Grade (1 to 4), where applicable. ^a^Measured Day -10; ^b^Measured Day 18; ^c^Measured Day 4.

On Day 1, one placebo- and three atacicept-treated patients had C3 levels below the LLN (Table 3). C3 and C4 levels rose during treatment in all patients, but started and remained below the LLN in Patient #14 (on atacicept), and were below the LLN at Day 1 and at end of treatment in Patient #2 (on placebo), having risen above the LLN during treatment. C3 levels were within the normal range at end-of-treatment assessments in the remaining three patients on atacicept and one on placebo (Table 3).

## Discussion

This study was terminated as a result of the emergence of unexpected, profound and rapid declines in IgG levels and the occurrence of serious infections. Several factors complicate the interpretation of the results. First, the large decreases in IgG began with initiation of MMF and high-dose CS two weeks before the initiation of atacicept (and continued IgG decrease). This was unexpected: to the authors' knowledge, such large decreases in IgG levels do not appear to have been reported in previous publications discussing MMF and/or CS treatment in patients with LN [5,6]. However, high-dose CS therapy has been reported to decrease IgG synthesis: in a patient with SLE, serum IgG levels fell from 1,580 to 630 mg/100 ml following 6 weeks' CS treatment (60 mg/day) [7]. In addition, hypogammaglobulinemia has been reported in approximately half of renal transplant patients receiving MMF in combination with CS [8]. It is of interest to note that an increased risk of infection has been observed in other LN studies during the induction phase with MMF and high-dose CS, compared with the maintenance phase [9,10]. The results reported here raise the possibility that this increased risk may be associated with severe hypogammaglobulinemia.

A second factor is that, by chance, those patients who were subsequently randomized to atacicept had higher proteinuria at screening (and through to Day 14) than those subsequently randomized to placebo, and all atacicept-randomized patients had UPr:Cr ratios ≥ 3.0 mg/mg at screening. This could have had a significant impact on the extent of IgG decrease. The magnitude of the decrease (up to 57% in the two weeks prior to atacicept initiation) suggests that the mechanism(s) for this decrease included an abnormally high excretion of IgG as well as a decrease in IgG production (associated with MMF and CS administration). This hypothesis is supported by the fact that proteinuria levels > 2 g/day are associated with the excretion of large proteins in the urine, which could include IgG. The high levels of proteinuria may have also affected the pharmacokinetics of atacicept, since atacicept is a protein that may be secreted in the urine in the setting of heavy proteinuria. Baseline GFR was, however, lower in the patients who were randomized to atacicept and, thus, the net effect of higher proteinuria and reduced GFR on atacicept secretion is not known.

In this study, lymphopenia and low CD4^+ ^counts were observed in both patients who developed serious pneumonia. Lymphocyte counts were low in all patients at baseline, before MMF initiation; this could be due to active lupus, as well as CS use. Indeed, lymphopenia and low IgG levels may each have been related to CS use, but were not necessarily related to each other. In clinical studies of atacicept to date, including healthy volunteers and patients with rheumatoid arthritis or generalized SLE, no association between atacicept and reduced T-cell, neutrophil or lymphocyte counts has been observed [11-13].

Atacicept inhibits both APRIL and BLyS, and has been shown to decrease levels of IgG, IgM and IgA, as well as naive B cells and plasma cells [11-13]. In Phase II studies in patients with rheumatoid arthritis, atacicept 150 mg was associated with median IgG decreases of approximately 30%. This decrease was near maximal by 12 to 16 weeks, no patients on atacicept had IgG levels below 3 g/l, and the rate of serious infections was not notably increased [11-13]. The results reported here are substantially different, suggesting that the biology of the disease likely contributed to this effect, and requires further study; the results may reflect alterations in proteins as yet unidentified in active LN.

## Conclusions

Increased risk of serious infections and large decreases in IgG were observed in patients with active LN and nephrotic-range proteinuria, in whom MMF and high-dose CS were initiated two weeks prior to atacicept (150 mg, twice weekly). Future studies will be needed to characterize the pharmacokinetic-pharmacodynamic, safety and efficacy profiles of atacicept in LN patients.

## Abbreviations

anti-dsDNA: antibodies against double-stranded DNA; APRIL: a proliferation-inducing ligand; BLyS: B-lymphocyte stimulator; CTX: cyclophosphamide; CS: corticosteroids; EOT: end of treatment; GFR: glomerular filtration rate; hpf: high-powered field; IgA: immunoglobulin A; IgG: immunoglobulin G; IgM: immunoglobulin M; LLN: lower limit of normal; LN: lupus nephritis; MMF: mycophenolate mofetil; RBCs: red blood cells; RR: reference range; SLE: systemic lupus erythematosus; ULN: upper limit of normal; UPr:Cr: urine protein:creatinine ratio; WBCs: white blood cells.

## Competing interests

EMG was a consultant to EMD Serono Inc.; SW is an employee of EMD Serono Inc.; AR and SC are employees of Merck Serono S.A.; JH is, and ER was, employed by ZymoGenetics Inc., Seattle, USA (a Bristol-Myers Squibb company); NGS was awarded funding for an unrelated investigator-initiated project from Merck USA. EMD Serono Inc., Merck Serono S.A. and Merck USA are all affiliates of Merck KGaA, Darmstadt, Germany.

## Authors' contributions

EMG participated in the development of the protocol, review and interpretation of the data, manuscript preparation and review. SW contributed to data collection and interpretation, manuscript development and finalization. AR reviewed and interpreted the data, and reviewed the manuscript during its development. SC participated in the statistical analysis of the data, review and interpretation of the data, and review of the manuscript. JH served as Medical Director in the trial, reviewed and interpreted the data, and reviewed the manuscript. ER provided oversight of the study design and participated in the writing and review of the manuscript for accuracy. NGS contributed to data collection and interpretation, and manuscript preparation. All authors read and approved the manuscript for publication.

## References

[B1] Singh S Saxena R Lupus nephritis Am J Med Sci 2009 337 451 460 10.1097/MAJ.0b013e3181907b3d 19390431

[B2] Tucci M Stucci S Strippoli S Silvestris F Cytokine overproduction, T-cell activation, and defective T-regulatory functions promote nephritis in systemic lupus erythematosus J Biomed Biotechnol 2010 2010 457146 20671931 10.1155/2010/457146 PMC2910555

[B3] Roschke V Sosnovtseva S Ward CD Hong JS Smith R Albert V Stohl W Baker KP Ullrich S Nardelli B Hilbert DM Migone TS BLyS and APRIL form biologically active heterotrimers that are expressed in patients with systemic immune-based rheumatic diseases J Immunol 2002 169 4314 4321 12370363 10.4049/jimmunol.169.8.4314

[B4] Arnett FC Edworthy SM Bloch DA McShane DJ Fries JF Cooper NS Healey LA Kaplan SR Liang MH Luthra HS The American Rheumatism Association 1987 revised criteria for the classification of rheumatoid arthritis Arthritis Rheum 1988 31 315 324 10.1002/art.1780310302 3358796

[B5] Ginzler EM Dooley MA Aranow C Kim MY Buyon J Merrill JT Petri M Gilkeson GS Wallace DJ Weisman MH Appel GB Mycophenolate mofetil or intravenous cyclophosphamide for lupus nephritis N Engl J Med 2005 353 2219 2228 10.1056/NEJMoa043731 16306519

[B6] Zhu B Chen N Lin Y Ren H Zhang W Wang W Pan X Yu H Mycophenolate mofetil in induction and maintenance therapy of severe lupus nephritis: a meta-analysis of randomized controlled trials Nephrol Dial Transplant 2007 22 1933 1942 10.1093/ndt/gfm066 17405792

[B7] McMillan R Longmire R Yelenosky R The effect of corticosteroids on human IgG synthesis J Immunol 1976 116 1592 1595 944738

[B8] Broeders EN Wissing KM Hazzan M Ghisdal L Hoang AD Noel C Mascart F Abramowicz D Evolution of immunoglobulin and mannose binding protein levels after renal transplantation: association with infectious complications Transpl Int 2008 21 57 64 17883369 10.1111/j.1432-2277.2007.00556.x

[B9] Appel GB Contreras G Dooley MA Ginzler EM Isenberg D Jayne D Li LS Mysler E Sánchez-Guerrero J Solomons N Wofsy D Aspreva Lupus Management Study Group Mycophenolate mofetil versus cyclophosphamide for induction treatment of lupus nephritis J Am Soc Nephrol 2009 20 1103 1112 10.1681/ASN.2008101028 19369404 PMC2678035

[B10] Wofsy D Appel GB Dooley MA Ginzler EM Isenberg D Jayne D Solomons N Lisk L The ALMS Study Group Aspreva Lupus Management Study maintenance results. The 9th International Congress on SLE June 2427 2010, Vancouver, Canada. CS12.6 and PO2.E.23 Lupus 2010 19 27 doi:10.1177/09612033100190010101 10.1177/0961203309345778 19933722

[B11] Dall'Era M Chakravarty E Wallace D Genovese M Weisman M Kavanaugh A Kalunian K Dhar P Vincent E Pena-Rossi C Wofsy D Reduced B lymphocyte and immunoglobulin levels after atacicept treatment in patients with systemic lupus erythematosus: results of a multicenter, phase Ib, double-blind, placebo-controlled, dose-escalating trial Arthritis Rheum 2007 56 4142 4150 10.1002/art.23047 18050206

[B12] Genovese MC Kinnman N de La Bourdonnaye G Pena Rossi C Tak PP Atacicept in patients with rheumatoid arthritis and an inadequate response to tumor necrosis factor antagonist therapy: results of a phase II, randomized, placebo-controlled, dose-finding trial Arthritis Rheum 2011 63 1793 1803 10.1002/art.30373 21452293

[B13] van Vollenhoven RF Kinnman N Vincent E Wax S Bathon J Atacicept in patients with rheumatoid arthritis and inadequate response to methotrexate: results of a phase II, randomized, placebo-controlled trial Arthritis Rheum 2011 63 1782 1792 21452294 10.1002/art.30372

